# Highly quantitative serological detection of anti-cytomegalovirus (CMV) antibodies

**DOI:** 10.1186/1743-422X-6-45

**Published:** 2009-05-01

**Authors:** Peter D Burbelo, Alexandra T Issa, Kathryn H Ching, Maurice Exner, W Lawrence Drew, Harvey J Alter, Michael J Iadarola

**Affiliations:** 1 Neurobiology and Pain Therapeutics Section, Laboratory of Sensory Biology, National Institute of Dental and Craniofacial Research, National Institutes of Health, Bethesda, Maryland 20892, USA; 2 Focus Diagnostics, Inc, Cypress, California 90630-4717, USA; 3 University of California, San Francisco, UCSF Medical Center at Mount Zion, San Francisco, California 94115, USA; 4 Infectious Disease Section, Department of Transfusion Medicine, NIH Clinical Center, National Institutes of Health, Bethesda, Maryland 20892, USA

## Abstract

**Background:**

Human cytomegalovirus infection is associated with a variety of pathological conditions including retinitis, pneumonia, hepatitis and encephalitis that may be transmitted congenitally, horizontally and parenterally and occurs both as a primary infection and as reactivation in immunocompromised individuals. Currently, there is a need for improved quantitative serological tests to document seropositivity with high sensitivity and specificity.

**Methods:**

Here we investigated whether luciferase immunoprecipitation systems (LIPS) would provide a more quantitative and sensitive method for detecting anti-CMV antibodies. Four protein fragments of immunodominant regions of CMV antigens pp150 and pp65 were generated as *Renilla *luciferase (Ruc) fusion proteins and used in LIPS with two cohorts of CMV positive and negative sera samples previously tested by ELISA.

**Results:**

Analysis of the antibody responses to two of these antigen fragments, pp150-d1 and pp150-d2, revealed geometric mean antibody titers in the first cohort that were 100–1000 fold higher in the CMV positive sera compared to the CMV negative samples (p < 0.0001) and infection status exactly matched the ELISA results for the 46 samples of the first cohort (100% sensitivity and 100% specificity). Two additional antigen fragments, pp65-d1 and pp65-d2 also showed robust antibody titers in some CMV-infected sera and yielded 50% and 96% sensitivity, respectively. Analysis of a second cohort of 70 samples using a mixture of the 4 antigens, which simplifies data collection and analysis, yielded values which correlated well with the sum of the values from the 4 separate tests (*r*_*s *_= 0.93, p < 0.00001). While comparison of the LIPS results from this second cohort with ELISA showed 100% sensitivity, LIPS detected six additional CMV positive samples that were not detected by ELISA. Heat map analysis revealed that several of the LIPS positive/ELISA negative samples had positive LIPS immunoreactivity with 3–4 of the CMV antigens.

**Conclusion:**

These results suggest that LIPS provides a highly robust and quantitative method for studying anti-CMV antibodies and has the potential to more accurately document CMV infection than standard ELISA.

## Introduction

Cytomegalovirus (CMV) is the largest member of the herpesvirus family, with a genome of approximately 230 kb encoding 160 genes [1]. Like several other herpes viruses, CMV infection is widespread and its seroprevalence in some lower socioeconomic communities can be greater than 90% [2]. In the United States, approximately 60% of the adult population is infected with CMV [3]. In most cases, initial infection with CMV presents without any overt symptoms. After primary infection, CMV infection remains latent in the body for life, but can show sporadic episodes of lytic activation. In immunocompromised individuals, including HIV-infected patients, CMV infection and reactivation can lead to ocular infections, encephalitis, and hepatitis [4]. CMV infection is also a common cause of febrile illnesses and graft rejection in transplant patients [5] and transfusion can lead to primary infection or reactivation of the virus [6]. CMV infection likely plays a role in vascular injury [7] and a variety of neurological problems including Guillain Barré syndrome [4,8]. Moreover, unlike other herpes viruses, a large number of CD4+ and CD8+ T-lymphocytes are dedicated to controlling CMV infection and studies have shown that the levels of these CMV specific T cells may decline during aging and illness [9]. CMV reactivation predicts morbidity and mortality in the elderly [10-12], in immunocompromised patients [13-17] and even in younger, immunocompetent individuals [18]. Given that CMV infection plays an important role in the pathogenesis of many different human conditions, better and more accurate methods are needed to diagnose and monitor immune responses to this infection.

Currently quantitative PCR- and DNA-based tests are useful for diagnosis and determining viral load [19]. However, understanding complex individual host responses to CMV infection will require more sophisticated information on disease status or processes than provided by current serological tests. The most quantitative serological immunoassays available to detect anti-CMV antibodies are ELISAs that use whole cell viral CMV lysates or recombinant CMV proteins usually produced in bacteria [20-22]. ELISAs employing CMV viral protein lysates contain a heterogeneous mixture of antigenic and non-antigenic proteins and have the potential to show cross-immunoreactivity with other herpes virus proteins. CMV proteins produced in bacteria as recombinant antigens can yield potential false signals and high backgrounds due to immunoreactivity with *E. coli *contaminants. Furthermore, solid phase ELISAs employing either CMV viral protein lysates or recombinant proteins require serial dilutions for semi-quantitative evaluation of antibodies and miss many conformational epitopes resulting in a limited dynamic range of detection. A more complicated CMV avidity ELISA, requiring serial dilutions, is used to distinguish primary verses long-term infection in longitudinal samples, but has limited dynamic range [23].

In order to circumvent some of the problems with solid phase ELISAs, we developed a liquid phase luciferase immunoprecipitation systems (LIPS). This system utilizes mammalian cell-produced, recombinant *Renilla *luciferase fusion antigens for efficiently constructing and expressing target antigens and quantitatively evaluating antibody responses [24-30]. LIPS has shown improved diagnostic performance compared to existing immunoassays for detecting antibodies to a variety of infectious agents [24,28-30] and has a wide dynamic range of detection providing new tools to monitor drug treatment [30] and sub-stratify disease states [28]. More recently, LIPS has been shown to be superior to ELISA to detect and monitor antibodies to herpes simplex virus (HSV)-1 and HSV-2 [31]. In the present study, LIPS was evaluated for its diagnostic performance in detecting anti-CMV antibodies.

## Results

### LIPS profiling of antibodies to four immunodominant CMV antigen fragments

**Figure 1:**
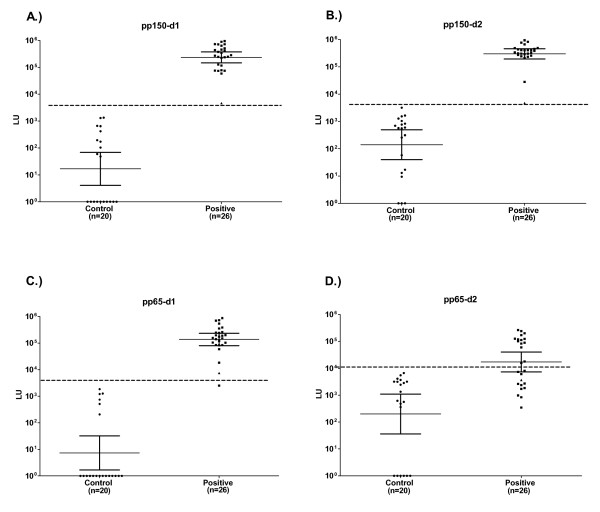
**Detection of anti-pp150-d1 (A), anti-pp150-d2 (B), anti-pp65-d1 (C), anti-pp65-d2 (D) antibodies by LIPS in the first sera cohort (n = 46)**. Each symbol represents individual samples from CMV-negative and CMV-positive subjects determined by ELISA. Antibody titers in LU are plotted on a log_10 _scale. The dashed line, derived from the mean plus five SD of the antibody titer of the 20 uninfected samples, serves as the cut-off level for determining sensitivity and specificity for each individual antigen test. The long solid horizontal lines indicate the GMT of the antibody in each group and the vertical lines show the 95% confidence intervals.

We generated four different immunodominant fragments of pp150 and pp65 as C-terminal *Renilla *luciferase (Ruc) fusion proteins using the pREN2 vector [25]. Previously described recombinant CMV protein fragments that were used [32] included two immunodominant fragments of pp150 spanning amino acids 502–692 (pp150-d1), and 859–1048 (pp150-d2) and two immunodominant fragments of pp65 spanning amino acids 2–295 (pp65-d1), and 312–561 (pp65-d2). These four constructs were then expressed in Cos1 cells and the lysates were used in the LIPS assay to evaluate a blinded sera cohort containing CMV seronegative and seropositive samples previously tested by ELISA. Following unmasking of the ELISA data, analysis of the geometric mean titer (GMT) for each of these antibody tests revealed that the CMV-positive sera had 800 to 2000-fold higher antibody titers compared to the CMV-negative sera (Figure 1). For example, in the CMV-negative sera the GMTs for pp150-d1, pp150-d2, pp65-d1 and pp65-d2 were 17; 140; 200; and 7 LU, respectively, while the GMTs in CMV-positive sera were markedly higher with values of 233,715; 297,680; 17,203; and 137,002 LU, respectively. Despite a wide range of titers, the results were highly reproducible. For example, the duplicate interassay LIPS tests for anti-pp150-d1 antibodies had a coefficient of variation (CV) of 14%. These results suggest that one benefit of the dynamic range of the LIPS format is that reproducible antibody titer differences of 100–1000-fold can be detected in the CMV-negative verses CMV-positive sera without the need for serial dilutions.

Receiver operator characteristics (ROC) analysis of each of the tests demonstrated that the anti-pp150-d1, anti-pp150-d2 and anti-pp65-d2 antibody tests had area under the curve (AUC) values of 1.0, reflecting the very high sensitivity and specificity of these tests. The anti-pp65-d2 antibody test was less useful with an AUC value of 0.84. Using a cut-off value derived from the mean plus five SD of the CMV negative samples, the pp150-d1 and pp150-d2 tests showed 100% sensitivity (26/26) and 100% specificity (20/20) for detecting CMV positive samples. Using this same cut-off criterion, the pp65-d1 test had 96% sensitivity (25/26) and 100% specificity (20/20), while the anti-pp65-d2 test demonstrated the least antigenicity with 54% sensitivity (14/26) and 100% specificity (20/20). Interestingly, a borderline CMV positive sample detected by ELISA was the single low positive outlier detected by LIPS (Figure 1).

**Figure 2:**
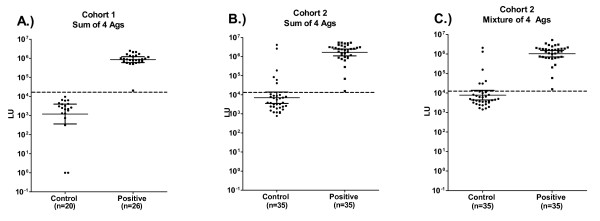
**CMV antibody titers to the sum of the four individual tests and using a mixture format**. Antibody titers to the sum of the 4 antigens in the first cohort (A), second cohort (B) or using a 4 antigen mixture format in the second cohort (C). Each symbol represents individual samples from CMV-negative and CMV-positive samples determined by ELISA. In the case of the first cohort, the sum of the titer values from the four individual tests and a cut-off value of 15,000, showed 100% sensitivity and 100% specificity. In the case of the second cohort, the LIPS tests from the sum of the 4 individual tests (B) or tested simultaneously as a mixture of four antigens (C) showed almost identical results; in each case, LIPS detected 6 samples that were ELSA negative. The solid horizontal lines indicate the GMT of the antibodies in each group and the vertical lines show the 95% confidence intervals.

In addition to analyzing the data from the four antigens separately and to compare to the mixture of antigens analyzed in a second, independent cohort (see below), we summed the antibody titers from the 4 individual tests and used a 15,000 LU cutoff, which was derived from the mean plus five standard deviations of the CMV negative samples. In this analysis, the 26 CMV positive samples detected in the individual tests were also detected in this combined approach and the 20 CMV negative samples again tested negative (Figure 2A). Together these results suggest that the single antigen tests (especially the pp150-d1 and pp150-d2 tests) and the combined results from the four individual tests provide an extraordinarily sensitive and specific method for profiling anti-CMV antibodies to diagnose infection.

### A four antigen mixture for profiling anti-CMV antibodies

Based on the results and cut-off values obtained in the first serum set, the four separate CMV LIPS tests were used with a new, second blinded cohort (n = 70 sera), which had been previously evaluated by ELISA. Prior to unblinding, we also analyzed these new sera for anti-CMV antibodies using a LIPS mixture format, where all four antigens were added together in a single well and processed simultaneously (Figure 2C). For comparison, the sum of the antibody titer results from the 4 separate antigen tests was also calculated and plotted (Figure 2B). As shown in Figure 2B and Figure 2C, the scatter plots of the sum titers of the 4 separate tests and the titer value of the four antigen mixture are almost identical. Regression analysis revealed that they closely correlated (Pearson *R *= 0.97, p < 0.00001). As in the individual antigen tests, the geometric means were over 100-fold greater in the CMV positive samples than the CMV negative samples. Using a cut-off value of 15,000 LU previously determined from cohort 1, both formats identified the same 41 potential positives and 29 potential negative samples (compare Figure 2B and 2C). Following unblinding, LIPS performance showed 100% sensitivity (35/35) and 83% specificity (29/35) in detecting the CMV infected sera in these two different LIPS assay formats. While these results do not exactly match the ELISA, the immunoreactivity profiles obtained from the individual LIPS tests matches that of the mixture format and further demonstrates the reproducibility and robust nature of this system.

**Figure 3:**
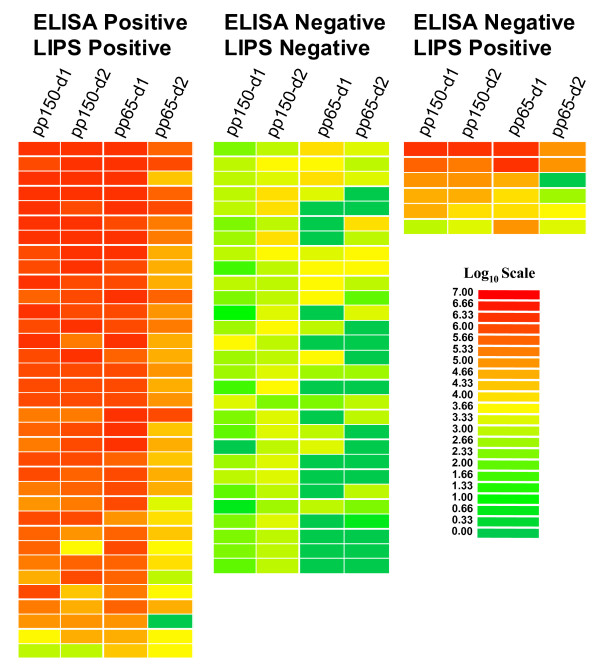
**Heat map representation of patient antibody profiles in second cohort to the four CMV antigens**. The titer values for each serum were log_10 _transformed and then the titer levels were color-coded as indicated by the log_10 _scale on the right, in which signal intensities range from green to red indicating low and high titers, respectively. The samples were rank ordered from highest to lowest based on the sum of the antibody titers to the four antigen panel. The samples on the left are from CMV infected sera and the samples in the middle panel represent the uninfected control sera. On the right are samples that were positive by the LIPS assay, but found to be negative according to the ELISA assay.

Log 10 transformed antibody titers and color coding were used to create a heatmap to easily visualize the different patient antibody responses toward the panel of antigens and to gain further insight into discordant samples (Figure 3). In this graphic, obvious marked differences in patient antibody responses to the antigen panel were observed, which illustrates the heterogeneity in individual humoral immune responses to the four individual CMV antigens (Figure 3). As shown in Figure 3, many of the ELISA positive/LIPS positive samples showed immunoreactivity to all four of the antigen fragments. One of the ELISA positive samples (Figure 3, bottom of heatmap of CMV positive samples) only showed positive LIPS immunoreactivity with the single anti-pp65-d1 antigen. Analysis of the discordant ELISA negative/LIPS positive samples showed that five of the six CMV ELISA negative samples had highly positive anti-pp150-d1 antibody titers. Furthermore, two of the ELISA negative samples were also positive by LIPS for anti-pp65-d2 and four samples were also positive for anti-pp65-d1 antibodies (Figure 3). It should be noted that many of these ELISA negative/LIPS positive samples showed markedly higher antibody titers than many of the samples that were positive by both ELISA and LIPS. Also, all of the discordant sera had low background binding reactivity with a lysate containing Ruc vector control protein suggesting that the observed immunoreactivity was not due to "sticky sera" or non-specific binding to the Renilla luciferase protein backbone (data not shown). Based on these results, it seems plausible that many of these discordant samples may represent true CMV positive samples that were missed by ELISA.

## Discussion

The use of LIPS allowed for highly quantitative measurements of antibody titers to 4 different CMV protein fragments. This simple modular assay system, where CMV antigens were expressed as a series of Ruc fusion proteins produced in Cos1 cells and then implemented in a liquid phase assay, efficiently evaluated patient humoral response to these different antigen fragments. Without serial dilution, the LIPS format showed titer differences spanning 100–1000 fold between the CMV-negative and CMV-positive sera samples. Immunoreactivity of the sera with multiple independent CMV antigen fragments, but not with control Ruc protein, strongly suggests that these samples contain anti-CMV antibodies detectable by LIPS. Our strategy of using 4 independent antigen fragments also allowed for independent assessment of antibodies against different protein fragments. In addition to the individual tests, we also used the LIPS assay in a mixture format to simultaneously test the four different recombinant antigens and acquire the data as a single read out. The ability to implement the test in a mixture format provides a simpler method of serologic detection. The fact that the four antigen mixture closely correlated with the data from the sum of the four individual antigen tests (R = 0.97) further demonstrates the reproducibility of the LIPS method and validates the results. The results from the mixture format are also consistent with our previous study of filarial infection where an antigen mixture was superior to ELISA in detecting antibodies to this infectious agent [30].

Compared to the ELISA, LIPS analysis of the two different cohorts yielded an overall detection rate of 100% sensitivity and 89% specificity. In the first cohort, compared to the ELISA, LIPS analysis yielded an overall detection rate of 100% sensitivity and 100% specificity. In the second cohort, six subjects in this cohort tested positive by LIPS, but were negative by ELISA. While this variability may be due to chance, it is important to note that five of these six samples were positive for three of the four CMV antigens, while the sixth sample was positive for only anti-pp65-d2 antibodies. One possible explanation for the discrepancy of CMV serological status between these two tests may lie in the different antigen sources used: the antigen source for the CMV ELISA is a complex viral lysate, which contains both antigenic and non-antigenic proteins that are coated to the microtiter plate, while the LIPS assay uses recombinant immunodominant CMV antigenic fragments of about 250 amino acids produced in mammalian cells and employed in a liquid phase assay. It is possible that ELISA negative/LIPS positive samples may represent early CMV infection, because immunodominant epitopes were used, which can detect low titer antibody from early infection better than ELISA using a crude lysate [30]. Therefore, it is possible if not probable, that these discordant samples represent true CMV positive samples that cannot be detected by ELISA. Consistent with this possibility, in another comparative study, LIPS detected anti-HSV-2 antibodies better than an ELISA and exactly matched the results of Western blot analysis [31]. Furthermore, several reports have shown that ELISA formats using viral lysates can miss CMV positive samples [8,32].

The ability to profile many different CMV antigens by this robust and facile approach may be useful for understanding host responses in different CMV-related diseases and for vaccine monitoring. In a previous study, LIPS detected relatively higher anti-HSV-2-specific glycoprotein antibodies in HSV-2 positive verses HSV-1 positive samples [31]. Based on a recent study that showed CMV strains could be distinguished by serology [33], LIPS screening of strain-specific glycoproteins from different CMV species might be a useful tool for genotyping studies. In sum, our current panel of four antigen fragments used either individually or as a mixture, have the potential to be highly useful tools to study anti-CMV antibody changes during the course of disease. Validation of this approach, with studies directed at monitoring CMV infection and reactivation following blood transfusion using longitudinal and larger sample numbers, are currently underway.

## Materials and methods

### Patient plasma

A first (n = 46 blinded sera) and second (n = 70) serum sets were provided as coded samples for testing with LIPS. The code was broken only after titers were established and categorization of CMV infection status had been made. Sera were kept at -80°C, aliquoted, and stored at 4°C. The sera were tested with IgG CMV Immunoassay (Focus Diagnostics, Cypress, CA) for CMV seropositive and seronegative status. The ELISA was considered the "standard" and sensitivity and specificity were determined based on ELISA results.

### Generation of Ruc-antigen fusion proteins

A mammalian *Renilla *luciferase (Ruc) expression vector, pREN2, was used to generate all plasmids. CMV protein fragments were amplified by PCR with gene specific linker-primer adapters: Four different protein fragments were amplified from CMV genomic DNA including pp150-d1 (aa 502–692), pp150-d2 (aa 859–1048), pp65-d1 (aa 2–295), and pp65-d2 (aa 312–561). In each case, the cDNA fragments were subcloned downstream of Ruc and a stop codon was inserted directly after the CMV protein coding sequence. The CMV sequence in each plasmid construct was confirmed by DNA sequencing. Details of the nucleotide and amino acid sequences can be found in the GenBank database with accession numbers FJ705802, FJ705803, FJ705804, and FJ705805 for pp150-d1, pp150-d2, pp65-d1, and pp65-d2, respectively. PCR primer sequences used to generate each construct are available upon request.

Fusion proteins for these four different protein fragments were generated by transfecting Cos-1 cells with individual Ruc expression vectors using Fugene-6. Forty-eight hours later the Cos1 cells were washed once with PBS and then scraped and sonicated on ice in lysis buffer (20 mM Tris, pH 7.5, 150 mM NaCl, 5 mM MgCl_2_, 1% Triton X-100 and 50% glycerol, and protease inhibitors (Complete Mini protease inhibitor cocktail tablets, Roche Diagnostics, Indianapolis, IN). The lysates were twice centrifuged at 13,000 × g, supernatants collected and then stored at -20°C until use. The activities of the lysates (light units (LU)/ml) were next determined using a single tube luminometer (20/20 from Turner Scientific) with a coelenterazine substrate mix (Promega, Madison, WI).

### LIPS analysis

LIPS assays were performed at room temperature using a 96-well plate format. Master plates were constructed by diluting patient plasma 1:10 in assay buffer A (20 mM Tris, pH 7.5, 150 mM NaCl, 5 mM MgCl_2_, 1% Triton X-100) in 96-well polypropylene microtiter plates. To quantify antibody titers by LIPS, 40 μl of buffer A, 10 μl of diluted human plasma (1 μl equivalent), and 50 μl of 1 × 10^7 ^light units (LU) of Ruc-antigen Cos1 cell extract, diluted in buffer A, were added to each well of polypropylene plates and incubated for 1 hour at room temperature. Next, 7 μl of a 30% suspension of Ultralink protein A/G beads (Pierce Biotechnology, Rockford, Illinois, USA) in PBS was added to the bottom of each well of a 96-well filter HTS plate (Millipore, Bedford, Massachusetts). The 100 μl antigen-antibody reaction mixture was then transferred to filter plates and incubated for 1 hour at room temperature on a rotary shaker. Proteins bound to the protein A/G beads were washed 10 times with buffer A and twice with PBS using a BioMek FX work station (Beckman Coulter, Fullerton, California, USA) with an integrated vacuum manifold. After the final wash, LU were measured in a Berthold LB 960 Centro microplate luminometer (Berthold Technologies, Bad Wilbad, Germany) using coelenterazine substrate mix (Promega, Madison, Wisconsin, USA). All of the LU data shown represent the average of two independent experiments and have been corrected for background LU values of Ruc Cos-1 cell extract added to protein A/G beads, but not incubated with plasma.

For the four antigen mixture tests, the assay was modified slightly. In these tests, each of the 4 antigen extracts (1 × 10^7 ^LU per antigen) were added to each well and processed as described above.

### Statistical analysis

GraphPad Prism software (San Diego, California, USA) was used for statistical analyses, including evaluating test performance by area under the curve (AUC). Results for quantitative antibody titers between uninfected controls, CMV-positive samples were reported as the geometric mean ± the 95% confidence interval. Mann-Whitney *U *tests were used for comparison of antibody titers in different groups and the level of significance was set at *P *< 0.05. Correlations between different antibody titers were assessed by Spearman correlation coefficient. For the calculation of sensitivity and specificity, a simple statistically based cut-off limit for each antigen was derived from the mean value of the uninfected samples plus 5 standard deviations.

## Competing interests

The authors declare that they have no competing interests.

## Authors' contributions

HA and PB initially conceived of the study. PB, AI and KC analyzed the sera by LIPS. LD and ME provided the sera samples used in this study and ELISA data on the samples. PB analyzed the data and drafted the manuscript. MI funded the study. All authors read and approved the manuscript.
